# Anchoring a Plant Cytochrome P450 via PsaM to the Thylakoids in *Synechococcus* sp. PCC 7002: Evidence for Light-Driven Biosynthesis

**DOI:** 10.1371/journal.pone.0102184

**Published:** 2014-07-15

**Authors:** Lærke Münter Lassen, Agnieszka Zygadlo Nielsen, Carl Erik Olsen, Wojciech Bialek, Kenneth Jensen, Birger Lindberg Møller, Poul Erik Jensen

**Affiliations:** 1 Center for Synthetic Biology “bioSYNergy”, the VILLUM Research Center “Plant Plasticity”, Copenhagen Plant Science Center, Department of Plant and Environmental Sciences, University of Copenhagen, Frederiksberg C, Copenhagen, Denmark; 2 Department of Biophysics, Faculty of Biotechnology, University of Wroclaw, Wroclaw, Poland; University of Hyderabad, India

## Abstract

Plants produce an immense variety of specialized metabolites, many of which are of high value as their bioactive properties make them useful as for instance pharmaceuticals. The compounds are often produced at low levels in the plant, and due to their complex structures, chemical synthesis may not be feasible. Here, we take advantage of the reducing equivalents generated in photosynthesis in developing an approach for producing plant bioactive natural compounds in a photosynthetic microorganism by functionally coupling a biosynthetic enzyme to photosystem I. This enables driving of the enzymatic reactions with electrons extracted from the photosynthetic electron transport chain. As a proof of concept, we have genetically fused the soluble catalytic domain of the cytochrome P450 CYP79A1, originating from the endoplasmic reticulum membranes of *Sorghum bicolor*, to a photosystem I subunit in the cyanobacterium *Synechococcus* sp. PCC 7002, thereby targeting it to the thylakoids. The engineered enzyme showed light-driven activity both *in vivo* and *in vitro*, demonstrating the possibility to achieve light-driven biosynthesis of high-value plant specialized metabolites in cyanobacteria.

## Introduction

Sunlight is an inexhaustible and abundant source of clean energy that, through photosynthesis, can be harvested by plants, algae and cyanobacteria. The amount of light energy captured in photosynthesis is often larger than what can be used by the photosynthetic metabolic reactions. This suggests a potential for utilizing photosynthetic organisms in biotechnology, as the excess electrons generated in photosynthesis could be applied in sustainable production of biofuels and high-value chemical compounds [1]. Using a synthetic biology approach, a photosynthetic organism can be engineered to take advantage of this excess reducing power. By transferring enzymes that catalyse reactions of interest into the vicinity of the thylakoids, it is possible to tap directly into the photosynthetic electron transport chain and re-direct some of the electrons towards production of valuable bioactive compounds [1], [2].

Plants synthesize a vast array of bioactive molecules, many of which have properties that make them useful as pharmaceuticals, biopesticides, flavours, fragrances or colours [3], [4]. Examples include paclitaxel from *Taxus brevifolia* and vincristine and vinblastine from *Catharanthus roseus*, which are all used in cancer treatment, and the anti-malarial agent artemisinin from *Artemisia annua* [5]–[7]. Often these compounds are of high market value, but are synthesized at low levels in the plants and are difficult to produce chemically due to their highly complex structures [4], [8], [9]. A group of enzymes often involved in the biosynthesis pathways of these molecules, catalysing key steps with high regio- and stereospecificity, is the cytochrome P450s (P450s). P450s are typically monooxygenases, catalysing the cleavage of O_2_ and incorporation of an oxygen atom in a wide variety of different substrates [10]–[12]. In plants, P450s are mainly found in the membranes of the endoplasmic reticulum (ER) and receive the electrons needed for their reactions from NADPH via a NADPH-cytochrome P450 oxidoreductase (POR) [13].

In this report it is our aim to construct a microbial *in vivo* production system for plant specialized metabolites, in which the POR is omitted and instead the electrons for the P450s are supplied from the photosynthetic electron transport chain.

In oxygenic photosynthesis, electrons are extracted from water by photosystem II (PSII) and delivered to photosystem I (PSI) via an electron transport chain [14], [15]. PSI utilizes energy from light to excite electrons and transfers them to ferredoxin (Fd), a small soluble redox protein, which in turn reduces proteins of different electron-requiring processes in the stroma of the chloroplast or cytosol of cyanobacteria, primarily the ferredoxin-NADP^+^ reductase (FNR), which catalyses the reduction of NADP^+^ to NADPH. The structure and function of PSI is highly conserved between organisms, the core of the complex even being remarkably conserved between cyanobacteria and higher plants despite a billion years of separate evolution [16], [17]. In addition, the high stability and quantum yield of PSI make it useful in bio-engineered production systems [18].

As a model system to investigate the effect of functionally coupling PSI and a plant P450, we have chosen the P450 CYP79A1 from the biosynthesis pathway of the cyanogenic glucoside dhurrin (β-D-glucopyranosyloxy-(S)-*p*-hydroxymandelonitrile) in the crop grass *Sorghum bicolor*. The dhurrin biosynthesis pathway consists of two membrane-anchored P450s, CYP79A1 and CYP71E1, and a soluble UDP-glucosyltransferase, UGT85B1. CYP79A1 is the first enzyme in the pathway and converts the substrate tyrosine into *p*-hydroxyphenylacetaldoxime (oxime) [19], [20].

We have previously shown that Fd is capable of mediating electron transfer from PSI directly to the CYP79A1 *in vitro*, thereby enabling oxime production with high efficiency [13]. The versatility of the approach of light-driven P450 reactions was demonstrated by showing that the system was not only functional with the plant CYP79A1, but also with a bacterial P450, the CYP124 from *Mycobacterium tuberculosis* [21]. More recently, we transiently expressed the entire dhurrin biosynthesis pathway in the tobacco *Nicotiana benthamiana*, where it was targeted to the chloroplast by transit peptides [2]. The two P450s were inserted in the thylakoid membrane and demonstrated to be driven by the photosynthetic reducing power.

In the present study we aimed at developing an *in vivo*-functional light-driven microbial production system by stably expressing a fusion protein of the catalytic domain of CYP79A1 and a PSI subunit, functioning as a thylakoid-targeting membrane anchor, in a cyanobacterium. Using a photosynthetic microorganism as host organism provides the opportunity for future scale-up to industrial production levels.

**Figure 1:**
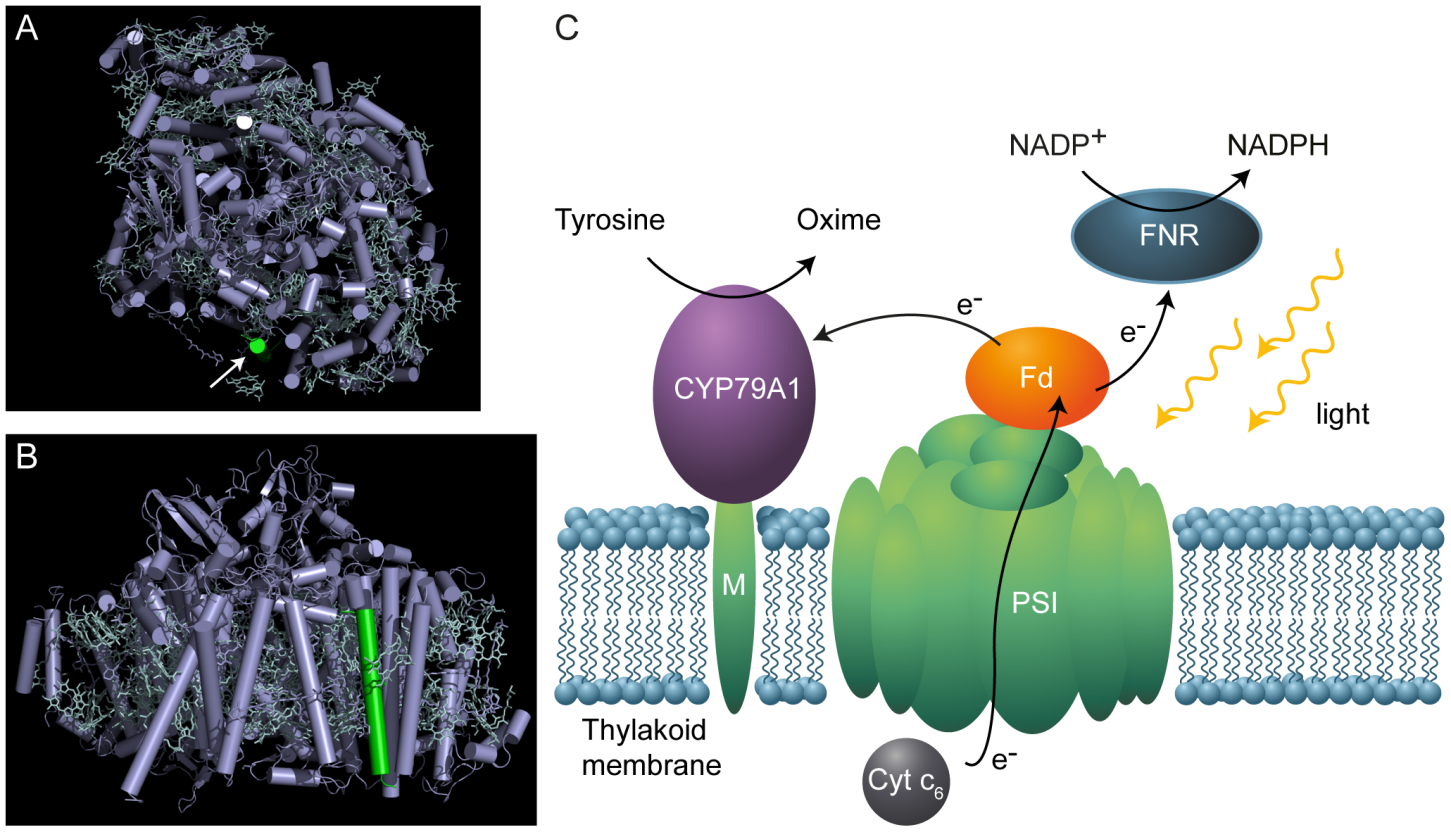
Localization of PsaM in PSI and schematic drawing of the thylakoid in the engineered strain. A) and B) The crystal structure of a monomer of photosystem I from the cyanobacterium *Thermosynechococcus elongatus* seen from the cytoplasmic side of the thylakoid membrane (A) and from the trimer-facing side of the monomer (B). RCSB Protein Data Bank ID: 1JB0. The PsaM subunit is shown in green in both panels and additionally indicated by an arrow in panel A. C) Schematic representation of the PsaM-CYP79A1 fusion protein in the thylakoid membrane.

For the cyanobacterium to be viable, only a part of the electrons from PSI should be delivered to the CYP79A1 via freely diffusible Fd. The peripheral, membrane embedded PSI subunit PsaM was chosen as anchor point for the CYP79A1 (Figure 1), as this should allow access of Fd to its docking site at PSI, even if the recombinant PsaM-CYP79A1 subunit remained bound to PSI in a PSI-CYP79A1 supercomplex, and ensure partition of electrons to both the CYP79A1 as well as to other proteins relying on reduction by Fd [22]. Proximity of the CYP79A1 to PSI should be beneficial in the competition for reduced Fd, as close confinement has been shown to be an advantage for the efficient electron transfer between interacting proteins in crowded environments like the thylakoids [23].

The PsaM-CYP79A1 fusion protein was shown to hydroxylate tyrosine to the corresponding oxime both *in vivo* and *in vitro*. Despite the fact that only a fraction of the fusion protein was found to be associated with PSI upon purification, the recombinant P450 was active without its native membrane anchor and its activity was indeed sustained by the photosynthetic electrons. Interestingly, the produced oxime was excreted into the growth medium, facilitating simple product isolation.

## Materials and Methods

### Construction of the *psaM-CYP79A1* fusion

A *psaM-CYP79A1* fusion construct was designed as a DNA sequence encoding amino acid 1-25 of *Synechococcus* sp. PCC 7002 PsaM, corresponding to the membrane spanning part of the protein, followed by a sequence encoding amino acid 36-558 of the *S. bicolor* CYP79A1, corresponding to the part of the CYP79A1 protruding from the membrane in the native protein, codon optimized for expression in cyanobacteria. The construct was commercially synthesized and inserted in the multi cloning site of the pUC57 cloning vector by Genscript (http://www.genscript.com/).

Genomic DNA (gDNA) was prepared from *Synechococcus* sp. PCC 7002 as described by Bickley and Owen (1995) [24]. The 831 bp flanking region upstream and 791 bp flanking region downstream of the *psaM* gene were amplified from the *Synechococcus* sp. PCC 7002 gDNA using primer pair b and c, respectively, and the *npt* cassette, conferring kanamycin resistance, was amplified from the pJHJ08 vector ([25], gift from associate professor Yumiko Sakuragi) using primer pair a by PCR (primers are listed in Table 1). The fragments were cloned into the pUC57 construct in three consecutive cloning steps at EcoRI, BamHI and BspEI sites as illustrated in Figure S1 to yield the *psaM-CYP79A1* fusion construct.

**Table 1:** Sequences of oligonucleotide primers used in this study.

Primer name	Sequence	Restriction site
a_Fwd_	5′-GCGTCCGGACCGGAATTGCCAGCTGGG-3′	BspEI
a_Rev_	5′-GCGGGATCCTCAGAAGAACTCGTCAAGAA-3′	BamHI
b_Fwd_	5′-GCGGAATTCGTCACAAAACCTGCATCTTC-3′	EcoRI
b_Rev_	5′-GCGGAATTCAATTTTAAAAACTCCGAATATAAAC-3′	EcoRI
c_Fwd_	5′-GCGGGATCCTAAATCTGCCACAGAAGCAG-3′	BamHI
c_Rev_	5′-GCGGGATCCTTTGTGTATCACACGCCTGA-3′	BamHI
d_Fwd_	5′-GGAAACAGGCAAGGTCTGTA-3′	-
d_Rev_, g_Rev_, h_Rev_	5′-GATTTGGGTCAGTTTGCGAC-3′	-
e_Fwd_, f_Fwd_	5′-ATGGGAATTTCTGATACCCAAG-3′	-
e_Rev_	5′-GAGAGCCGGAAAGCCAAA-3′	-
f_Rev_	5′-CGCAGGTGGTGGAACTAC-3′	-
g_Fwd_	5′-GTTATCCGGCGTCTCATGTT-3′	-
h_Fwd_	5′-GACTGGGCACAACAGACAAT-3′	-

Restriction sites inserted into the PCR product through the primers are underlined.

### Transformation of cyanobacteria

Transformation of *Synechococcus* sp. PCC 7002 with the fusion construct to obtain the PsaM-CYP79A1 strain was performed as described by Frigaard *et al.* (2004) [26]. The *psaM* gene was replaced with the *psaM-CYP79A1* gene through homologous recombination. Transformants were selected for kanamycin resistance and segregated for 4 generations before they were screened for the presence of the fusion gene by colony PCR using primer pairs d, g and h (Table 1).

### Cyanobacterial strains and culture conditions

Two *Synechococcus* sp. PCC 7002 strains were used in this study, wild type (WT) and the recombinant PsaM-CYP79A1 strain. Cyanobacterial cells were grown photoautotrophically in medium A^+^ (medium A [27] containing 1 mg/mL NaNO_3_) at 38°C under continuous illumination at 80 µmol·m^−2^·s^−1^ with aeration by 3% (v/v) CO_2_ in air or on A^+^ agar plates containing 15 g/L Bacto agar. PsaM-CYP79A1 engineered cells were grown in the presence of 200 µg/mL kanamycin. Cell growth was monitored by measuring the absorbance at 730 nm (optical density, OD_730_). The chlorophyll (Chl) content was measured after extraction with 80% acetone as described by Lichtenthaler [28]. Cultures for experiments and isolation of thylakoids were started at OD_730_  = 0.05. The cells were harvested when they were in the late exponential phase as assessed by measuring the OD_730_.

### RNA isolation and RT-PCR

RNA was isolated from 20 mL *Synechococcus* sp. PCC 7002 cultures grown to the late exponential phase. The cells were harvested by centrifugation at 10,000×g for 3 min and the pellets were resuspended in 100 µL double-distilled water. 100 µL glass beads and 800 µL TRIzol reagent (Invitrogen) were added and the samples were subjected to 6 rounds of freezing in liquid nitrogen, thawing and 1 min vigorous vortexing. After 5 min incubation at room temperature (RT), 200 µL chloroform was added and the samples were vortexed for 15 s, and incubated at RT for 3 min. The phases were separated by centrifugation at 12,000×g for 10 min at 4°C and the upper phases transferred to new tubes. 500 µL isopropanol was added and the samples were incubated for 10 min at RT, and centrifuged at 12,000×g for 10 min at 4°C. The pellets were washed in 1 mL 70% ethanol by vortexing and centrifugation at 7,500×g for 5 min at 4°C. The supernatants were discarded and the pellets dried for 15 min before they were dissolved in 100 µL RNase-free water. The RNA was purified using the RNeasy Plant Mini Kit (Qiagen) and the concentrations were measured to be 128 ng/µL for the WT and 202 ng/µL for the PsaM-CYP79A1 using a NanoDrop ND-1000 Spectrophotometer (Thermo Scientific).

The RNA was prepared for RT-PCR (reverse transcriptase polymerase chain reaction) using the DNase I Amplification Grade Kit (Sigma-Aldrich). cDNA was synthesized from the RNA using the iScript cDNA Synthesis Kit (Bio-Rad) with the primer mixture supplied with the kit (a mixture of oligo(dT) and random hexamer primers). A control sample was made without reverse transcriptase. The cDNA was used as template for PCR using primer pairs e and f (Table 1).

### *In vivo* PsaM-CYP79A1 activity

40 mL cultures of WT and PsaM-CYP79A1 were grown as described above. Cells were separated from the growth medium by centrifugation at 5,000×g for 10 min at 4°C. Metabolites were extracted from the growth medium by vortexing with 1/3 volume of ethyl acetate (EtOAc) three times. The cells were disrupted by sonication (20 cycles of 30 s pulse amplitude 100, 90 s off) in a Q700 sonicator (Qsonica) equipped with a cup horn. Samples were centrifuged at 10,000×g for 10 min at 4°C and metabolites were extracted from the supernatant into a double volume of EtOAc by thorough vortexing. For both growth medium and cell samples, the EtOAc phase was separated from the water phase by centrifugation at 2,000×g for 5 min. The EtOAC was evaporated in a Scanspeed 32 (Scanvac, Labogene). The samples were resuspended in 80% methanol (MeOH), diluted to 20% MeOH in water and subjected to liquid chromatography–mass spectrometry (LC-MS) analysis (final volume 80 µL).

### LC-MS analysis

Analytical LC-MS was carried out using an Agilent 1100 Series LC (Agilent Technologies, Germany) coupled to a Bruker HCT-Ultra ion trap mass spectrometer (Bruker Daltonics, Bremen, Germany). A Zorbax SB-Aq column (Agilent; 3.5 µM, 2.1×150 mm) was used at a flow rate of 0.2 mL min^−1^. The oven temperature was maintained at 35°C. The mobile phases were: A, 2 mM ammonium acetate; B, methanol. The gradient program was: 0 to 1 min, isocratic 25% B; 1 to 11 min, linear gradient 25 to 60% B; 11 to 12 min, isocratic 98% B; 12 to 20 min, isocratic 25% B. The mass spectrometer was run in positive APCI mode and the recorded mass range was m/z 80-350.

### Isolation of thylakoids and PSI from cyanobacteria

All steps were carried out in green safelight or dim light with samples kept in the dark at 4°C or on ice. The cells were collected by centrifugation at 7,500×g for 10 min at 4°C and washed once in 20 mM Tricine-NaOH pH 7.5. The cells were then pelleted again, resuspended in thylakoid buffer (20 mM Tricine-NaOH pH 7.5, 0.4 M sucrose, 10 mM NaCl, 5 mM MgCl_2_, 1 mM DTT, 5% (v/v) glycerol, 1× Complete protease inhibitor cocktail (Roche)) and disrupted in a single cycle at 20,000 psi in a TS Series Benchtop cell disrupter (Constant Systems). Unbroken cells were removed by centrifugation at 3,000×g for 10 min at 4°C and the supernatant was subjected to ultracentrifugation at 184,000×g for 45 min at 4°C. The pellet, consisting of the thylakoids, was carefully resuspended in thylakoid buffer using a small paintbrush. If not to be used for isolation of PSI, the thylakoids were frozen in liquid nitrogen.

For isolation of PSI, the thylakoids were solubilised in 1% (w/v) *n*-dodecyl β-D-maltoside (β-DM) at a final concentration corresponding to 1 mg Chl/mL (dilution in thylakoid buffer) by stirring for 30 min on ice. Unsolubilised membranes were removed by ultracentrifugation at 184,000×g for 30 min at 4°C. Supernatant corresponding to 0.5 mg Chl was applied to 5-step 10–30% sucrose gradients prepared as described by Chitnis and Chitnis (1993) [29]. The sucrose gradients were ultracentrifuged at 270,000×g for 21 hours at 4°C and green bands were carefully collected using a Pasteur pipette or 0.5 mL fractions were collected using a peristaltic pump.

### *In vitro* PsaM-CYP79A1 activity measurements

Thylakoids corresponding to 100 µg Chl were mixed with 2.6 µM (0.25 µCi) L-[U-^14^C] Tyrosine (Perkin Elmer) or 0–1000 µM unlabelled tyrosine (Sigma-Aldrich), 0.15 mM spinach Fd, 22.5 µM *Synechococcus* sp. PCC 7002 cytochrome c_6_ (cyt c_6_) expressed in *Escherichia coli*, 3.6 mM sodium ascorbate, 0.11 mM 2,6-dichlorophenolindophenol (DCPIP), 1 mM dithiothreitol (DTT) and 0.1% (w/v) β-DM in a 20 mM Tricine-NaOH buffer pH 7.5 (total reaction volume 200 µL). Assay samples where incubated at 25°C while illuminated with a Schott KL 1500 light source at 200 µmol·m^−2^·s^−1^ for 30 min. The produced oxime was extracted in EtOAc corresponding to twice the reaction volume by vortexing. The EtOAc phase was separated from the assay mixture by centrifugation at 2,000×g for 5 min, transferred to a new tube and concentrated in a Scanspeed 32 vacuum concentrator. In experiments using radiolabelled tyrosine as substrate, the sample components were separated by thin layer chromatography (TLC) using a toluene/EtOAc/MeOH (30∶8∶1 v/v) solvent mixture and a Silica gel 60 F_254_ TLC plate (Merck). A dilution series of L-[U-^14^C] Tyrosine was included on the plate to allow quantification. The TLC plate was incubated with a storage phosphor screen for 2 weeks and radiolabelled components were detected and quantified using a Storm 840 PhosphorImager (Molecular Dynamics). In the experiments where unlabelled tyrosine was used as substrate, two samples were prepared for each data point and the EtOAc pooled afterwards. Samples were prepared for and subjected to LC-MS analysis as described above.

### Immunoblot analysis

Thylakoid (proteins corresponding to 10 µg Chl) and PSI (proteins corresponding to 50 µg Chl) or sucrose gradient fraction samples (proteins from 80 µL of the 0.5 mL fractions collected from the sucrose gradient, concentrated by acetone precipitation by centrifugation at 20,000×g for 15 min at 4°C in a final concentration of 80% acetone) were subjected to SDS-PAGE on 12% Criterion XT Bis-Tris gels (Bio-Rad) in XT MES Running Buffer (Bio-Rad) at 200 V for 55 min. Proteins were transferred to a Whatman Protran nitrocellulose membrane using a Criterion blotter (Bio-Rad). The membrane was blocked in 5% (w/v) skimmed milk in PBS buffer containing 0.05% (v/v) Tween-20 (PBS-T) for 1 h at RT and incubated overnight with a rabbit CYP79A1 primary antibody (1∶2000 dilution, gift from Tomas Laursen) in 1% (w/v) skimmed milk in PBS-T at 4°C. For the sucrose gradient fraction immunoblot, the membrane was cut in three after the blocking step and the pieces were incubated with the CYP79A1 primary antibody, a rabbit PsaC primary antibody (1∶1000 dilution, Agrisera) and a rabbit PsbA primary antibody (1∶10,000 dilution, Agrisera), respectively. The blot was washed with PBS-T and incubated with a secondary horseradish peroxidase (HRP)-conjugated swine anti-rabbit IgG antibody (Dakopatts, 1∶5000 dilution in PBS-T) for 1 h at RT. The membrane was washed again in PBS-T and the secondary antibody detected using SuperSignal West Dura Chemiluminescent Substrate (Pierce) with a BioSpectrum Imaging System (UVP).

### BN-PAGE analysis

Blue-native polyacrylamide gel electrophoresis (BN-PAGE) was performed by a procedure adapted from the laboratory of Prof. Jörg Nickelsen, Ludwig Maximilians University of Munich [30]. Briefly, thylakoids corresponding to 500 µg protein were pelleted by centrifugation at 20,000×g for 20 min at 4°C and resuspended in 50 µL ACA buffer (50 mM Bis-Tris, pH 7.0, 750 mM aminocaproic acid, 0.5 mM EDTA). The thylakoids were solubilized by adding 7.5 µL 10% (w/v) β-DM and incubating on ice for 30 min. Samples were centrifuged at 20,000×g for 30 min at 4°C and supernatant was mixed with 8 µL loading buffer (750 mM aminocaproic acid, 5% (w/v) Coomassie G-250). 25 µL sample was loaded on a NativePAGE Novex 4–16% Bis-Tris Protein Gel (Life Technologies). Gel electrophoresis was performed at 4°C by first running at 40 V constant for 7 h with blue catode buffer (50 mM tricine, 15 mM Bis-Tris, pH 7.0, 0.2% (w/v) Coomassie G-250) and clear anode buffer (50 mM Bis-Tris, pH 7.0), then at 300 V constant in clear catode buffer (50 mM tricine, 15 mM Bis-Tris, pH 7.0) and clear anode buffer until reaching the end of the gel.

## Results

### Construction of a *psaM-CYP79A1* fusion DNA sequence for homologous recombination in cyanobacteria

A PsaM-CYP79A1 fusion construct was designed by replacing the native membrane-spanning N-terminal domain of the CYP79A1 with the PsaM subunit of cyanobacterial PSI. A fusion gene of *psaM* and a codon-optimized version of the sequence encoding the soluble domain of the CYP79A1 were commercially synthesized, and flanking regions of the *psaM* gene and a kanamycin resistance cassette were cloned into the construct (Figure S1).

The marine cyanobacterium *Synechococcus* sp. PCC 7002 was chosen as photosynthetic host organism. Its available genomic sequence and natural transformability facilitate genetic engineering. Additionally, being tolerant to a broad range of environmental conditions and capable of fast photoautotrophic growth with simple nutritional requirements, this cyanobacterium is a good candidate for use in biotechnological applications. *Synechococcus* sp. PCC 7002 cells were incubated with the DNA construct to allow uptake and the native *psaM* gene was replaced with the *psaM-CYP79A1* gene through homologous recombination.

### Verification of the presence of PsaM-CYP79A1 in transformed cyanobacteria

**Figure 2:**
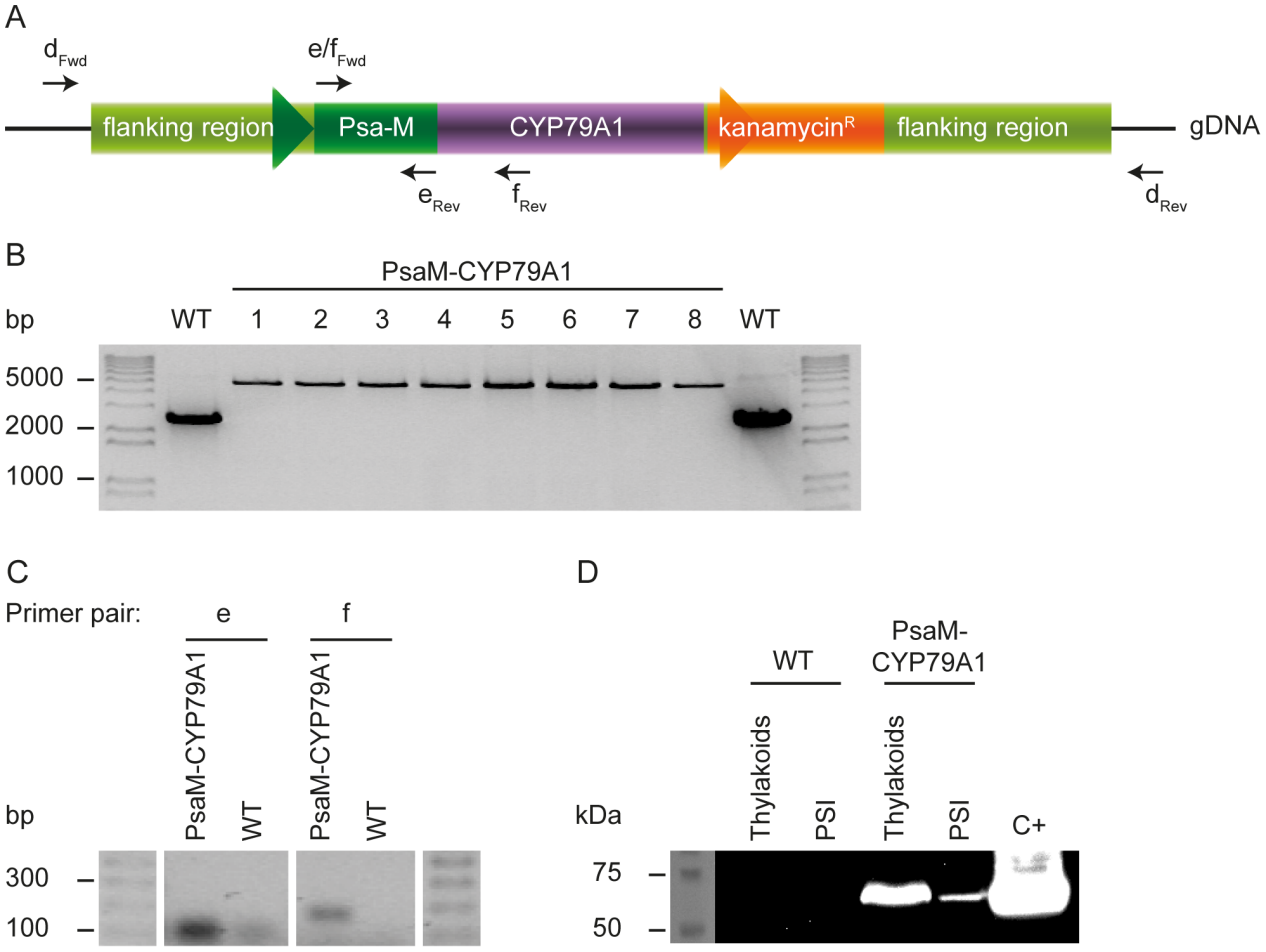
Gene construct and verification of transformation. A) Schematic representation of binding sites for primers used for colony PCR (primer pair d, subpanel B) and RT-PCR (primer pair e and f, subpanel C) in the transformed *Synechococcus* sp. PCC 7002 genomic DNA. The black lines represent the gDNA that is not part of the cloning construct. The same forward primer is used in primer pair e and f. The primers are shown in Table 1. B) Colony PCR analysis of WT and transformed *Synechococcus* sp. PCC 7002 colonies performed using primer pair d. The sizes of the bands correlate well with the expected sizes of 4841 bp (PsaM-CYP79A1 strain) and 2344 bp (WT) indicating that the fusion construct has replaced the native *psaM* gene in the transformed strain. C) RT-PCR analysis of the transcription of the *psaM-CYP79A1* fusion gene. The sizes of the fragments fit the expected 77 bp for primer pair e (expected in both WT and PsaM-CYP79A1) and 142 bp for primer pair f (only expected for PsaM-CYP79A1). D) Immunoblot of WT and transformant thylakoid (proteins corresponding to 10 µg Chl) and trimeric PSI (proteins corresponding to 50 µg Chl) samples with an anti-CYP79A1 antibody. C+: CYP79A1 expressed in chloroplasts of tobacco. The expected molecular masses of the CYP79A1 with its native membrane anchor (C+) and the PsaM-CYP79A1 are ∼62 kDa and ∼61 kDa, respectively, deduced from the corresponding DNA sequences.

Transformants were segregated for 4 generations using kanamycin as positive selection marker, and colony PCR was performed to confirm that the fusion gene construct had been integrated in the *Synechococcus* sp. PCC 7002 genome. Using primers binding outside the recombined sequence, it was verified that a DNA construct with a size corresponding to the *psaM*-*CYP79A1* fusion gene and the kanamycin resistance cassette had replaced the native *psaM* gene in the transformed strain (Figure 2 A and B). Only one band was seen for each colony of the transformed strain, thus indicating that *psaM* had been replaced by the fusion construct in all copies of the genome. Moreover, when the forward primer binding site was located in the *CYP79A1* part of the fusion gene or in the kanamycin resistance cassette, colony PCR products were only obtained for the transformed strain (data not shown).

RT-PCR was used to investigate if the *psaM-CYP79A1* fusion DNA was transcribed in the cyanobacteria (Figure 2A and C). RNA was purified from WT and transformed cultures and used for cDNA synthesis in the presence and absence of reverse transcriptase. PCR using primer pair e with both the forward and reverse primer binding in the *psaM* part of the fusion gene resulted in a product in both WT and PsaM-CYP79A1 samples. When primer pair f with the reverse primer binding in the *CYP79A1* part of the fusion gene was used, only the sample with the transformed strain resulted in a PCR product. In control PCR samples run in the absence of reverse transcriptase, no bands were seen (data not shown).

The presence of expressed PsaM-CYP79A1 fusion protein in thylakoids and trimeric PSI purified from the transformed *Synechococcus* sp. PCC 7002 was analysed by immunoblotting. As positive control, full-length CYP79A1 (∼62 kDa) expressed in *Nicotiana benthamiana* chloroplasts was used. A band of approximately the same size, corresponding to the expected size (∼61 kDa) of the fusion of PsaM and the soluble domain of the CYP79A1, was observed in both thylakoids and trimeric PSI of the PsaM-CYP79A1 cyanobacteria, whereas no signal was detected in the WT samples, thus indicating that the PsaM-CYP79A1 fusion was expressed in the transformed cyanobacteria (Figure 2D) and present in at least a fraction of the trimeric PSI complexes.

### PsaM-CYP79A1 localization

**Figure 3:**
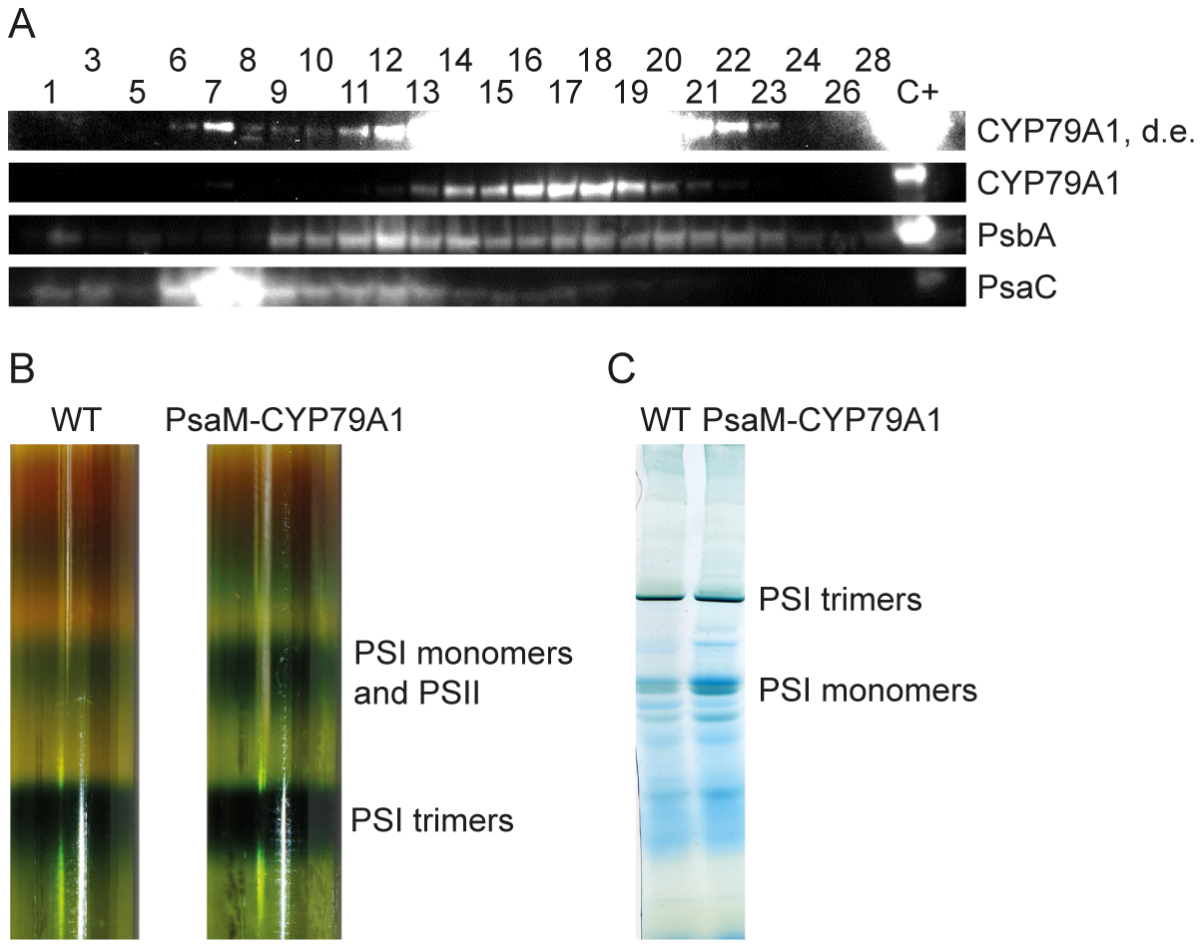
Localization of the PsaM-CYP79A1 fusion protein in the thylakoids. A) Immunoblot of proteins from 80 µL of the 0.5 mL fractions collected from the PsaM-CYP79A1 sucrose gradient shown in panel B), concentrated through acetone precipitation. Antibodies against the PSI subunit PsaC (∼9 kDa), the PSII subunit PsbA (∼32 kDa) and the CYP79A1 (∼61 kDa) have been used. CYP79A1, d.e.: digitally enhanced representation of the chemiluminescence signal detected from the CYP79A1 bands (shown below). C+: CYP79A1 expressed in chloroplasts of tobacco. Fraction 1 is the bottommost and fraction 28 the topmost fraction in the gradient. B) Sucrose gradients separating the components of WT and PsaM-CYP79A1 thylakoids solubilized in 1% (w/v) β-DM. C) Separation of protein complexes of β-DM-solubilized WT and PsaM-CYP79A1 thylakoids by BN-PAGE.

Having established that the fusion protein is expressed we analysed the localisation of the protein in more detail. In the immunoblot analysis in Figure 2D, the samples contained thylakoids and PSI trimers corresponding to 10 and 50 µg Chl, respectively. The thylakoid sample thus contained much less PSI complexes than the PSI trimer sample. A stronger signal was clearly detected from the thylakoid sample, indicating that a large part of the PsaM-CYP79A1 fusion present in the thylakoids is not associated with the PSI trimers after purification. This was confirmed in an independent experiment by analysing the presence of PSI, PSII and CYP79A1 by immunoblot analysis of representative fractions of a sucrose gradient separating solubilized membrane components of the thylakoids using antibodies against the PSI-subunit PsaC, the PSII-subunit PsbA and the CYP79A1 (Figure 3). While a small fraction of the PsaM-CYP79A1 co-migrated with the PSI trimers, most PsaM-CYP79A1 was detected in fractions of the sucrose gradient shifted towards smaller complexes than the photosynthetic protein complexes (Figure 3A). This indicates that the PsaM-CYP79A1 fusion is not firmly attached to the PSI trimers nor monomers. It is possible that the PsaM-CYP79A1 fusion is associated with PSI in the intact membranes, but most PsaM-CYP79A1 is not bound to PSI upon detergent-mediated solubilisation of the thylakoid membrane.

An increase in the abundance of the PSI monomer band was observed in the sucrose gradient with thylakoids from the PsaM-CYP79A1 strain compared to the gradient prepared from wild type thylakoids (Figure 3B). This increase in PSI monomers in the engineered strain was likewise verified by BN-PAGE (Figure 3C).

### *In vivo* PsaM-CYP79A1 activity

**Figure 4:**
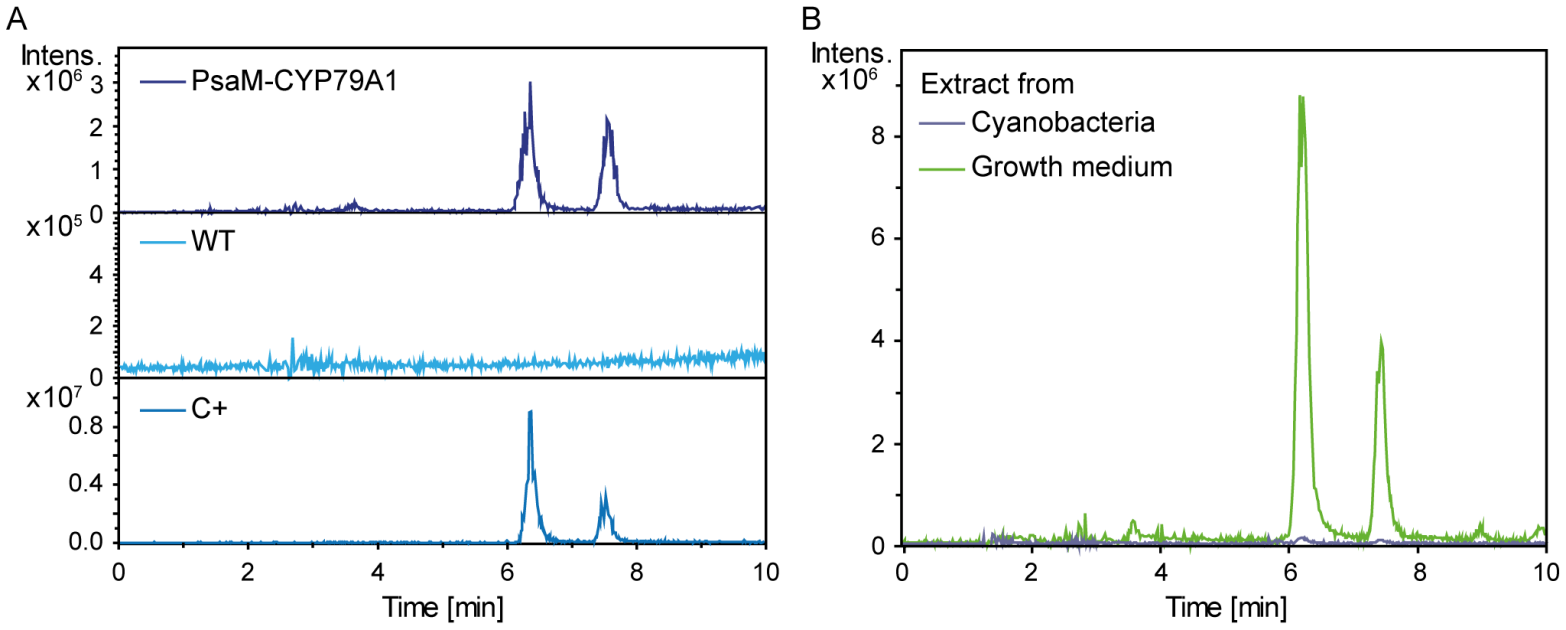
Verification of product formation in living cells. A) Extracted ion chromatograms (*m/z* 152) from the LC-MS analysis of metabolites extracted from the growth medium of *Synechococcus* sp. PCC 7002 WT and PsaM-CYP79A1 cultures for detection of *in vivo* activity of the PsaM-CYP79A1 complex, compared to a *p*-hydroxyphenylacetaldoxime standard (C+). The two peaks are the E and Z isomers of the *p*-hydroxyphenylacetaldoxime. B) LC-MS extracted ion chromatograms as in A, from analysis of extracts of PsaM-CYP79A1 cyanobacteria or growth medium.

The *in vivo* enzymatic activity of CYP79A1 in the engineered PsaM-CYP79A1 strain was investigated by analysing extracts of cyanobacteria cells and growth medium by LC-MS. WT and PsaM-CYP79A1 cultures were grown to an OD_730_ of 2.5 before they were harvested and metabolites from the cyanobacteria cells and the growth medium were extracted in ethyl acetate and methanol. The samples were analysed by LC-MS in APCI mode. Extracted ion chromatograms (EIC) were created corresponding to [M+H]^+^ (*m*/*z* 152) for *p*-hydroxyphenylacetaldoxime. The results are shown in Figure 4.

Only the PsaM-CYP79A1 samples contained a product with retention times identical to those of the oxime standard, thus clearly demonstrating that the engineered cyanobacterial strain can convert tyrosine into the corresponding oxime and that the PsaM-CYP79A1 fusion is enzymatically active *in vivo* in cyanobacteria (Figure 4A). Interestingly, the oxime was detected almost exclusively in the growth medium, implying that the product is excreted from the cyanobacteria (Figure 4B). At OD_730_  =  2.5, the oxime content was found to be approximately 4 ng/mL culture in the growth medium of the PsaM-CYP79A1 strain. The expression and enzymatic activity of the PsaM-CYP79A1 fusion protein in the cyanobacteria had no apparent impact on growth of the cyanobacteria cells compared with the wild type (data not shown).

### PsaM-CYP79A1 activity in isolated thylakoids

Having demonstrated that the PsaM-CYP79A1 fusion enzyme was active *in vivo*, we now turned to *in vitro* studies with purified thylakoids. Enzymatic activity assays were performed to verify that the oxime production by the PsaM-CYP79A1 fusion indeed was light-driven. Isolated thylakoids were incubated with tyrosine (radiolabelled or unlabelled) in the presence of electron donors for PSI and Fd for mediation of electron transport from PSI to the CYP79A1 [2], [13]. The activity assay was performed under illumination in the presence of detergent to disrupt the thylakoids and allow access of the added electron donors to the oxidizing site of PSI. After completion of the assay, hydrophobic metabolites were extracted from the assay mixture with ethyl acetate. The components of the samples prepared with radiolabelled tyrosine as substrate were separated by TLC and analysed by autoradiography using storage phosphor screens. Unlabelled tyrosine samples were analysed by LC-MS. Oxime produced in the assays was identified through comparison with an oxime standard.

**Figure 5:**
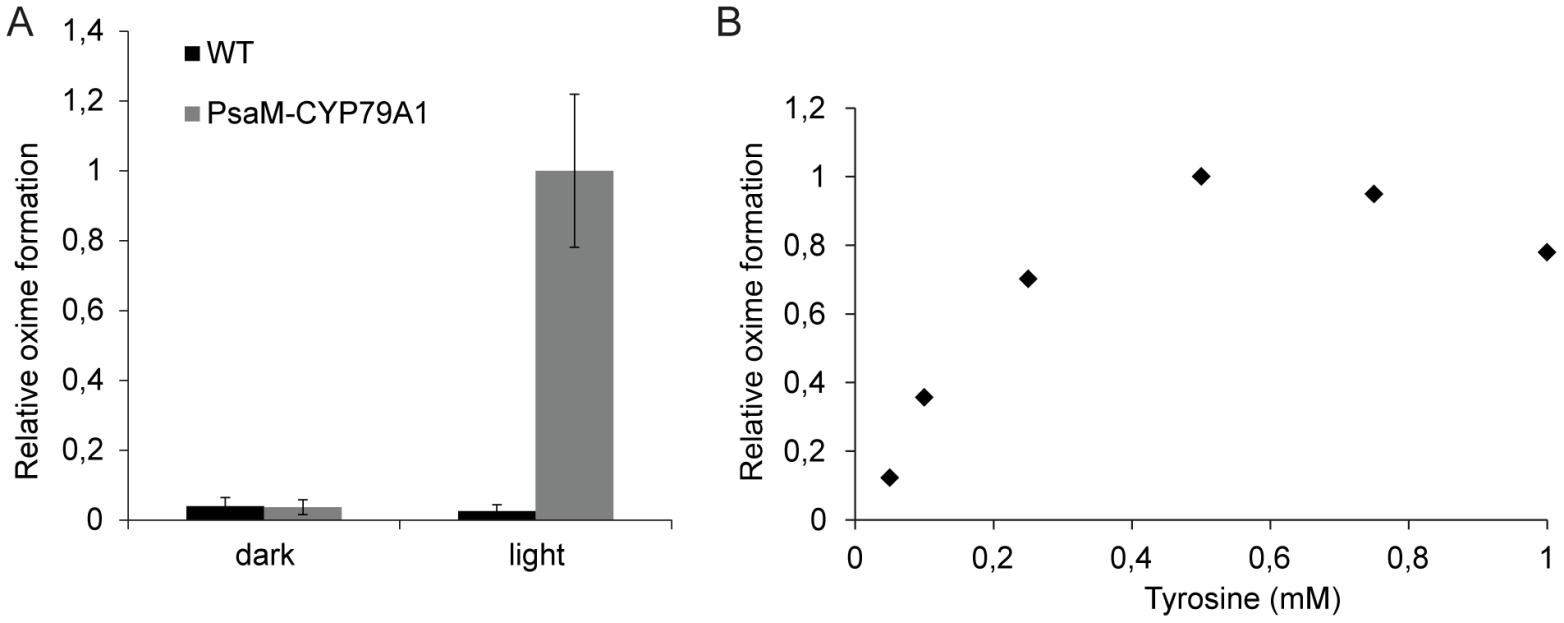
Evidence for light-driven CYP79A1 activity. *In vitro* PsaM-CYP79A1 enzyme activity assays. A) Using radiolabelled tyrosine as substrate, assays were run using WT and PsaM-CYP79A1 thylakoids incubated at 25°C for 30 min under 200 µmol·m^−2^·s^−1^ illumination or in the dark. The produced radiolabelled oxime was detected by laser scanning of a storage phosphor screen incubated with the TLC plate on which the extracted cyanobacterial metabolites were separated. The data represent averages of 5 measurements and error bars show standard deviations. B) PsaM-CYP79A1 thylakoids were assayed as in A), but with unlabelled tyrosine, and metabolite extracts were analysed by LC-MS. The result from a typical experiment is shown.

Figure 5A shows the *in vitro* formation of radiolabelled oxime by WT and PsaM-CYP79A1 thylakoids in the light or in the dark. Only the PsaM-CYP79A1 thylakoids were able to produce oxime. Oxime formation was only seen in light-incubated samples with a 27 fold increase in signal compared to dark-incubated samples, thereby demonstrating that the oxime formation was light-driven and indicating that the PsaM-CYP79A1 was indeed fuelled with electrons delivered from PSI.

Figure 5B shows the oxime production in PsaM-CYP79A1 thylakoids as a function of tyrosine concentration. An increase in the production level was seen up to a concentration of 0.5 mM tyrosine. At higher concentrations, the PsaM-CYP79A1 appears to be substrate inhibited. At a substrate concentration of 0.5 mM tyrosine, approximately 12 ng oxime was produced by thylakoids corresponding to 100 µg Chl during a 30 min assay.

## Discussion

### Light-driven oxime production by an engineered cyanobacterium

In this work, we have genetically linked the catalytic domain of the CYP79A1 from the plant *S. bicolor* to the PSI subunit PsaM in *Synechococcus* sp. PCC 7002 with the aim to target the CYP79A1 to the vicinity of PSI to obtain light-driven P450 biosynthesis. The PsaM-CYP79A1 fusion protein was found to be located in the thylakoids of the cyanobacterial host with a smaller fraction directly attached to the PSI complex. The fusion protein was functional *in vivo* with the CYP79A1 enzymatic activity being sustained by endogenously produced tyrosine and the product, *p*-hydroxyphenylacetaldoxime, being excreted into the growth medium (Figure 4). Through *in vitro* assays, the enzymatic reaction was confirmed to be light-driven, indicating that the electrons powering the PsaM-CYP79A1 catalytic cycle were indeed photosynthetic electrons delivered from PSI (Figure 5).

Cytochrome P450s are key enzymes in the biosynthesis of the majority of the numerous bioactive specialized metabolites with medicinal properties produced by plants. For many of these compounds, the biosynthesis in the plants is tightly regulated with the production levels often being low or highly variable, dependent on induction by abiotic or biotic factors and confined to specific growth stages and cell types, thus making extraction, purification and separation from structurally similar compounds a challenge [3], [4], [8], [9]. This study demonstrates that it is possible to express plant P450s in the thylakoids of a cyanobacterium to obtain a light-driven production system as an environmentally friendly alternative to production through chemical synthesis.

As the P450 fold is highly conserved, and since delivery of electrons from PSI to both the CYP79A1 from *S. bicolor* and the CYP124 from *M. tuberculosis* via Fd have been shown to function *in vitro*, it is probable that the light-driven biosynthesis approach is generally applicable to a variety of P450s [13], [21], [31].

In addition to uncoupling from the tight metabolic regulation that the enzyme is often subjected to in the native host, an advantage of heterologous expression in a microorganism is the potential for future scale-up of production to an industrial level. The commercial use of cyanobacterial production systems is still in its developing phase, but recently cyanobacteria have attracted interest due to their green bioindustrial potential. The simple nutritional growth requirements, constituting only water, CO_2_ and a few mineral nutrients, with sunlight as the only necessary energy source, suggest a potential for inexpensive and renewable production of for instance biofuels and valuable natural products. Cyanobacterium-based production systems thus offer an alternative to the carbohydrate-fed approaches necessary for the microorganisms primarily employed in biotechnology [32]–[35].

The oxime produced by the PsaM-CYP79A1 in this study was excreted into the growth medium, a phenomenon that has also earlier been observed for heterologous production of caffeic acid and the monoterpene β-phellandrene in the cyanobacterium *Synechocystis* sp. PCC 6803, and that with these compounds facilitate easy product isolation [36], [37]. However, more knowledge about the secretion mechanism is needed before we can take full advantage of this phenomenon.

### Electron partitioning and fusion construct design

For the cyanobacterium to be viable while supplying electrons for the PsaM-CYP79A1, electron delivery should be possible not only to the P450, but also to other processes in the thylakoid environment dependent on reduction by Fd. It was thus decided not to attempt to obtain direct electron transfer from the terminal Fe_4_S_4_ clusters of PSI to the CYP79A1, as has been the aim for *in vitro* coupling of hydrogenases or metal catalysts to PSI, but rather to rely on electron transfer via Fd [38]–[42]. It was chosen to anchor the CYP79A1 to the peripheral PsaM subunit of PSI where the P450 would be expected not to prevent access of Fd to its docking site at PSI [22]. Though most CYP79A1 appeared not to be directly associated with PSI, at least after purification, the engineered CYP79A1 was found to be located in the thylakoids and active without its native membrane anchor, demonstrating the possibility of engineering P450s without inactivating the enzymatic capability. This suggests that it may be possible to develop other P450 fusion proteins for this light-driven setup, possibly providing increased production levels through improved competitiveness for reduced Fd.

### Association of PsaM-CYP79A1 and PSI?

From the immunoblot analysis in Figure 2 and 3, the main part of the PsaM-CYP79A1 fusion protein was found not to co-fractionate with the PSI complex. It could be that most PsaM-CYP79A1 has never been associated with PSI, but it is also possible that the PsaM-CYP79A1 is loosely connected to PSI in the intact membranes, but that the complexes disintegrate upon membrane solubilisation. The added size of the CYP79A1 domain on the PsaM protein could result in steric hindrances and thereby weakened interactions between the PsaM and the remaining PSI complex.

The PsaM subunit has previously been found to stabilize, but not be strictly needed, for the assembly of PSI trimers in a *psaM* ^–^ mutant of *Synechocystis* sp. PCC 6803 [43]. The increased abundance of PSI monomers observed following expression of PsaM-CYP79A1 (Figure 3) could thus be explained by a reduction in the number of stable trimers due to the absence of the PsaM-CYP79A1 fusion protein in a majority of the PSI complexes. Alternatively, trimers containing the PsaM-CYP79A1 could be present in the membrane, but the inter-complex interactions weakened due to the CYP79A1, causing the instable trimers to fall apart during purification.

No impact on growth was detected for the PsaM-CYP79A1 strain compared to the WT despite the observed destabilization or reduced abundance of PSI trimers in the thylakoids of the PsaM-CYP79A1 strain. It is to be noted that normal growth was also observed in the *Synechocystis* sp. PCC 6803 *psaM* ^–^ mutant [43].

### Increasing the yield of light-driven biosynthesis

The compound produced by light-driven biosynthesis in this project, *p*-hydroxyphenylacetaldoxime, is chosen for proof-of-concept. However, for a compound produced for a commercial application, the product titer would have to be increased from the current production level of ng/mL culture to be of industrial relevance. Key parameters in this context will be optimization of the expression level of the enzymes in the pathway, the supply of substrate and delivery of electrons to the P450s. Upregulation of the expression of Fd may lead to an increased supply of electrons to the P450s, and engineering of the P450 and/or Fd to increase the affinity of the two proteins for each other may also increase the ability of the P450 to compete for reduced Fd with FNR. Another approach that earlier has proved to yield an increase in production for a P450 was co-expression of a cytochrome *b*_5_, which can stimulate the P450 reaction rate through several possible mechanisms [44], [45].

The flavodiiron proteins Flv1 and Flv3 have been shown to function as a sink for excess electrons produced in photosynthesis in cyanobacteria, reducing O_2_ to water without production of reactive oxygen species [46]. As the light-driven approach can also be seen as an additional electron sink, it could be speculated that knocking down those flavodiiron proteins would increase the need for an alternative sink and that this perhaps could lead to an increased supply of electrons to the P450s.

For a pathway containing multiple enzymes, the flux through the pathway will be important to optimize. This can be approached by adjusting the relative expression level of the enzymes, e.g. by changing promoters and ribosome-binding sites, and increasing the product channelling, which may be improved e.g. by scaffolding of the enzymes [47]–[49]. Balancing the protein expression can however be a challenge, as engineering of cyanobacteria is still not as well established as engineering of classic model microorganisms such as *E. coli*, which has a less complex metabolism, and even characterized genetic elements can result in unpredictable expression levels [50].

## Conclusion and Perspectives

In this study, we have engineered a fusion enzyme of the catalytic domain of a plant P450 and a PSI subunit and expressed it in *Synechococcus* sp. PCC 7002. The PsaM-CYP79A1 fusion enzyme is present in the thylakoids and proved to be functional with light-driven enzymatic activity detected both *in vivo* and *in vitro*. The biosynthesized oxime was found to be excreted into the growth medium, enabling easy product isolation. These works demonstrate the possibility of functionally coupling plant enzymes requiring electrons in their reactions to PSI and utilize the reducing equivalents generated in the photosynthetic electron transport chain to obtain light-driven biosynthesis in photosynthetic microorganisms.

It is envisioned that the approach presented here can be developed as a light-driven and environmentally friendly production system for bioactive compounds relevant to the pharmaceutical or chemical industries. In contrast to most enzymes used as biocatalysts *in vitro* for industrial purposes, P450s require the costly cofactor NADPH and a reductase for electron donation, an issue that is overcome in the light-driven *in vivo* system presented here [51]. Cyanobacteria can be efficiently cultivated at industrial scale and the use of a cyanobacterial host thus provides the potential for future scale-up of the system.

## Supporting Information

Figure S1 **Cloning of the PsaM-CYP79A1 fusion construct.** Using PCR, a kanamycin resistance cassette was amplified from a vector containing the cassette (*npt* cassette from pJHJ08, [25]) and flanking regions of the *psaM* gene were amplified from *Synechococcus* sp. PCC 7002 gDNA. Primer sequences are shown in Table 1. The PCR products were inserted in a commercially synthesized fusion construct of the *psaM* and *CYP79A1* genes contained in the pUC57 cloning vector. The final construct was transformed into *Synechococcus* sp. PCC 7002 for replacement of the native *psaM* gene by homologous recombination.
(TIF)

## References

[1] Lassen LM, Nielsen AZ, Ziersen B, Gnanasekaran T, Moller BL, et al (2014) Redirecting Photosynthetic Electron Flow into Light-Driven Synthesis of Alternative Products Including High-Value Bioactive Natural Compounds. Acs Synthetic Biology 3: 1–12. 24328185 10.1021/sb400136f

[2] Nielsen AZ, Ziersen B, Jensen K, Lassen LM, Olsen CE, et al (2013) Redirecting Photosynthetic Reducing Power toward Bioactive Natural Product Synthesis. Acs Synthetic Biology 2: 308–315. 23654276 10.1021/sb300128r

[3] Marienhagen J, Bott M (2012) Metabolic engineering of microorganisms for the synthesis of plant natural products. Journal of Biotechnology 163: 166–178. 22687248 10.1016/j.jbiotec.2012.06.001

[4] Balandrin MF, Klocke JA, Wurtele ES, Bollinger WH (1985) Natural plant chemicals: Sources of industrial and medicinal materials. Science 228: 1154–1160. 3890182 10.1126/science.3890182

[5] Rao SR, Ravishankar GA (2002) Plant cell cultures: Chemical factories of secondary metabolites. Biotechnology Advances 20: 101–153. 14538059 10.1016/s0734-9750(02)00007-1

[6] Cragg GM, Newman DJ (2005) Plants as a source of anti-cancer agents. Journal of Ethnopharmacology 100: 72–79. 16009521 10.1016/j.jep.2005.05.011

[7] Covello PS, Teoh KH, Polichuk DR, Reed DW, Nowak G (2007) Functional genomics and the biosynthesis of artemisinin. Phytochemistry 68: 1864–1871. 17399751 10.1016/j.phytochem.2007.02.016

[8] Chemler JA, Koffas MA (2008) Metabolic engineering for plant natural product biosynthesis in microbes. Current opinion in biotechnology 19: 597–605. 18992815 10.1016/j.copbio.2008.10.011

[9] Jensen K, Jensen PE, Moller BL (2012) Light-driven chemical synthesis. Trends in Plant Science 17: 533–539. 10.1016/j.tplants.2011.12.008 22306522

[10] Hannemann F, Bichet A, Ewen KM, Bernhardt R (2007) Cytochrome P450 systems - biological variations of electron transport chains. Biochimica Et Biophysica Acta-General Subjects 1770: 330–344. 10.1016/j.bbagen.2006.07.017 16978787

[11] Lamb DC, Waterman MR, Kelly SL, Guengerich FP (2007) Cytochromes P450 and drug discovery. Current opinion in biotechnology 18: 504–512. 18006294 10.1016/j.copbio.2007.09.010

[12] Werck-Reichhart D, Feyereisen R (2000) Cytochromes P450: a success story. Genome Biol 1: REVIEWS 3003.3001–3003.3009. 10.1186/gb-2000-1-6-reviews3003 PMC138896 11178272

[13] Jensen K, Jensen PE, Moller BL (2011) Light-Driven Cytochrome P450 Hydroxylations. Acs Chemical Biology 6: 533–539. 21323388 10.1021/cb100393j

[14] Amunts A, Nelson N (2009) Plant Photosystem I Design in the Light of Evolution. Structure 17: 637–650. 19446520 10.1016/j.str.2009.03.006

[15] Nelson N, Ben-Shem A (2004) The complex architecture of oxygenic photosynthesis. Nature Reviews Molecular Cell Biology 5: 971–982. 15573135 10.1038/nrm1525

[16] Amunts A, Nelson N (2008) Functional organization of a plant photosystem I: Evolution of a highly efficient photochemical machine. Plant Physiology and Biochemistry 46: 228–237. 18272382 10.1016/j.plaphy.2007.12.013

[17] Xu W, Tang HD, Wang YC, Chitnis PR (2001) Proteins of the cyanobacterial photosystem I. Biochimica Et Biophysica Acta-Bioenergetics 1507: 32–40. 10.1016/s0005-2728(01)00208-0 11687206

[18] Jensen PE, Bassi R, Boekema EJ, Dekker JP, Jansson S, et al (2007) Structure, function and regulation of plant photosystem I. Biochimica Et Biophysica Acta-Bioenergetics 1767: 335–352. 10.1016/j.bbabio.2007.03.004 17442259

[19] Bak S, Kahn RA, Nielsen HL, Moller BL, Halkier BA (1998) Cloning of three A-type cytochromes p450, CYP71E1, CYP98, and CYP99 from Sorghum bicolor (L.) Moench by a PCR approach and identification by expression in Escherichia coli of CYP71E1 as a multifunctional cytochrome p450 in the biosynthesis of the cyanogenic glucoside dhurrin. Plant Molecular Biology 36: 393–405. 9484480 10.1023/a:1005915507497

[20] Nielsen KA, Tattersall DB, Jones PR, Moller BL (2008) Metabolon formation in dhurrin biosynthesis. Phytochemistry 69: 88–98. 17706731 10.1016/j.phytochem.2007.06.033

[21] Jensen K, Johnston JB, de Montellano PRO, Moller BL (2012) Photosystem I from plants as a bacterial cytochrome P450 surrogate electron donor: terminal hydroxylation of branched hydrocarbon chains. Biotechnology Letters 34: 239–245. 21983973 10.1007/s10529-011-0768-4 PMC3671864

[22] Setif P, Fischer N, Lagoutte B, Bottin H, Rochaix JD (2002) The ferredoxin docking site of photosystem I. Biochimica Et Biophysica Acta-Bioenergetics 1555: 204–209. 10.1016/s0005-2728(02)00279-7 12206916

[23] Moal G, Lagoutte B (2012) Photo-induced electron transfer from photosystem I to NADP(+): Characterization and tentative simulation of the in vivo environment. Biochimica Et Biophysica Acta 1817: 1635–1645. 22683536 10.1016/j.bbabio.2012.05.015

[24] Bickley J, Owen RJ (1995) Preparation of bacterial genomic DNA. Methods in molecular biology (Clifton, NJ) 46: 141–147. 10.1385/0-89603-297-3:141 7550704

[25] Jacobsen JH, Rosgaard L, Sakuragi Y, Frigaard N-U (2011) One-step plasmid construction for generation of knock-out mutants in cyanobacteria: studies of glycogen metabolism in Synechococcus sp PCC 7002. Photosynthesis Research 107: 215–221. 21302031 10.1007/s11120-010-9613-1

[26] Frigaard NU, Sakuragi Y, Bryant DA (2004) Gene inactivation in the cyanobacterium Synechococcus sp. PCC 7002 and the green sulfur bacterium Chlorobium tepidum using in vitro-made DNA constructs and natural transformation. Methods Mol Biol 274: 325–340. 15187290 10.1385/1-59259-799-8:325

[27] Stevens SE, Patterso.Co, Myers J (1973) The production of hydrogen peroxide by blue-green algae: a survey Journal of Phycology. 9: 427–430.

[28] Lichtenthaler HK (1987) Chlorophylls and Carotenoids: Pigments of Photosynthetic Biomembranes. Methods in Enzymology 148: 350–382.

[29] Chitnis VP, Chitnis PR (1993) PsaL subunit is required for the formation of photosystem I trimers in the cyanobacterium Synechocystis sp. PCC 6803. Febs Letters 336: 330–334. 8262256 10.1016/0014-5793(93)80831-e

[30] Schottkowski M, Gkalympoudis S, Tzekova N, Stelljes C, Schuenemann D, et al (2009) Interaction of the Periplasmic PratA Factor and the PsbA (D1) Protein during Biogenesis of Photosystem II in Synechocystis sp PCC 6803. Journal of Biological Chemistry 284: 1813–1819. 19017636 10.1074/jbc.M806116200

[31] Hamdane D, Zhang H, Hollenberg P (2008) Oxygen activation by cytochrome P450 monooxygenase. Photosynthesis Research 98: 657–666. 18600471 10.1007/s11120-008-9322-1 PMC2743973

[32] Ducat DC, Way JC, Silver PA (2011) Engineering cyanobacteria to generate high-value products. Trends in Biotechnology 29: 95–103. 21211860 10.1016/j.tibtech.2010.12.003

[33] Parmar A, Singh NK, Pandey A, Gnansounou E, Madamwar D (2011) Cyanobacteria and microalgae: a positive prospect for biofuels. Bioresour Technol 102: 10163–10172. 21924898 10.1016/j.biortech.2011.08.030

[34] Rosgaard L, de Porcellinis AJ, Jacobsen JH, Frigaard NU, Sakuragi Y (2012) Bioengineering of carbon fixation, biofuels, and biochemicals in cyanobacteria and plants. Journal of Biotechnology 162: 134–147. 22677697 10.1016/j.jbiotec.2012.05.006

[35] Quintana N, Van der Kooy F, Van de Rhee MD, Voshol GP, Verpoorte R (2011) Renewable energy from Cyanobacteria: energy production optimization by metabolic pathway engineering. Applied Microbiology and Biotechnology 91: 471–490. 21691792 10.1007/s00253-011-3394-0 PMC3136707

[36] Bentley FK, García-Cerdán JG, Chen H-C, Melis A (2013) Paradigm of Monoterpene (β-phellandrene) Hydrocarbons Production via Photosynthesis in Cyanobacteria. BioEnergy Research 6: 917–929.

[37] Xue Y, Zhang Y, Grace S, He Q (2013) Functional expression of an Arabidopsis p450 enzyme, p-coumarate-3-hydroxylase, in the cyanobacterium Synechocystis PCC 6803 for the biosynthesis of caffeic acid. Journal of Applied Phycology: 1–8.

[38] Grimme RA, Lubner CE, Bryant DA, Golbeck JH (2008) Photosystem I/molecular wire/metal nanoparticle bioconjugates for the photocatalytic production of H-2. Journal of the American Chemical Society 130: 6308–6309. 18439011 10.1021/ja800923y

[39] Ihara M, Nishihara H, Yoon KS, Lenz O, Friedrich B, et al (2006) Light-driven hydrogen production by a hybrid complex of a [NiFe]-hydrogenase and the cyanobacterial photosystem I. Photochemistry and Photobiology 82: 676–682. 16542111 10.1562/2006-01-16-RA-778

[40] Krassen H, Schwarze A, Friedrich B, Ataka K, Lenz O, et al (2009) Photosynthetic Hydrogen Production by a Hybrid Complex of Photosystem I and [NiFe]-Hydrogenase. Acs Nano 3: 4055–4061. 19947646 10.1021/nn900748j

[41] Lubner CE, Knorzer P, Silva PJN, Vincent KA, Happe T, et al (2010) Wiring an FeFe -Hydrogenase with Photosystem I for Light-Induced Hydrogen Production. Biochemistry 49: 10264–10266. 21058656 10.1021/bi1016167

[42] Utschig LM, Dimitrijevic NM, Poluektov OG, Chemerisov SD, Mulfort KL, et al (2011) Photocatalytic Hydrogen Production from Noncovalent Biohybrid Photosystem I/Pt Nanoparticle Complexes. Journal of Physical Chemistry Letters 2: 236–241.

[43] Naithani S, Hou JM, Chitnis PR (2000) Targeted inactivation of the psaK1, psaK2 and psaM genes encoding subunits of Photosystem I in the cyanobacterium Synechocystis sp PCC 6803. Photosynthesis Research 63: 225–236. 16228433 10.1023/A:1006463932538

[44] Paddon CJ, Westfall PJ, Pitera DJ, Benjamin K, Fisher K, et al (2013) High-level semi-synthetic production of the potent antimalarial artemisinin. Nature 496: 528–536. 23575629 10.1038/nature12051

[45] Schenkman JB, Jansson I (2003) The many roles of cytochrome b(5). Pharmacology & Therapeutics 97: 139–152. 12559387 10.1016/s0163-7258(02)00327-3

[46] Helman Y, Tchernov D, Reinhold L, Shibata M, Ogawa T, et al (2003) Genes encoding a-type flavoproteins are essential for photoreduction of O-2 in cyanobacteria. Current Biology 13: 230–235. 12573219 10.1016/s0960-9822(03)00046-0

[47] Dueber JE, Wu GC, Malmirchegini GR, Moon TS, Petzold CJ, et al (2009) Synthetic protein scaffolds provide modular control over metabolic flux. Nat Biotech 27: 753–759. 10.1038/nbt.1557 19648908

[48] Holtz WJ, Keasling JD (2010) Engineering Static and Dynamic Control of Synthetic Pathways. Cell 140: 19–23. 20085699 10.1016/j.cell.2009.12.029

[49] Khosla C, Keasling JD (2003) Metabolic engineering for drug discovery and development. Nat Rev Drug Discov 2: 1019–1025. 14654799 10.1038/nrd1256

[50] Oliver JWK, Machado IMP, Yoneda H, Atsumi S (2014) Combinatorial optimization of cyanobacterial 2,3-butanediol production. Metabolic Engineering 22: 76–82. 24412567 10.1016/j.ymben.2014.01.001

[51] van Beilen JB, Duetz WA, Schmid A, Witholt B (2003) Practical issues in the application of oxygenases. Trends in Biotechnology 21: 170–177. 12679065 10.1016/S0167-7799(03)00032-5

