# Supplementary figures and images for: Anchoring a Plant Cytochrome P450 via PsaM to the Thylakoids in Synechococcus sp. PCC 7002: Evidence for Light-Driven Biosynthesis
Source: PLoS One. 2014 Jul 15;9(7):e102184. doi: 10.1371/journal.pone.0102184 (PMC4099078; doi:10.1371/journal.pone.0102184)

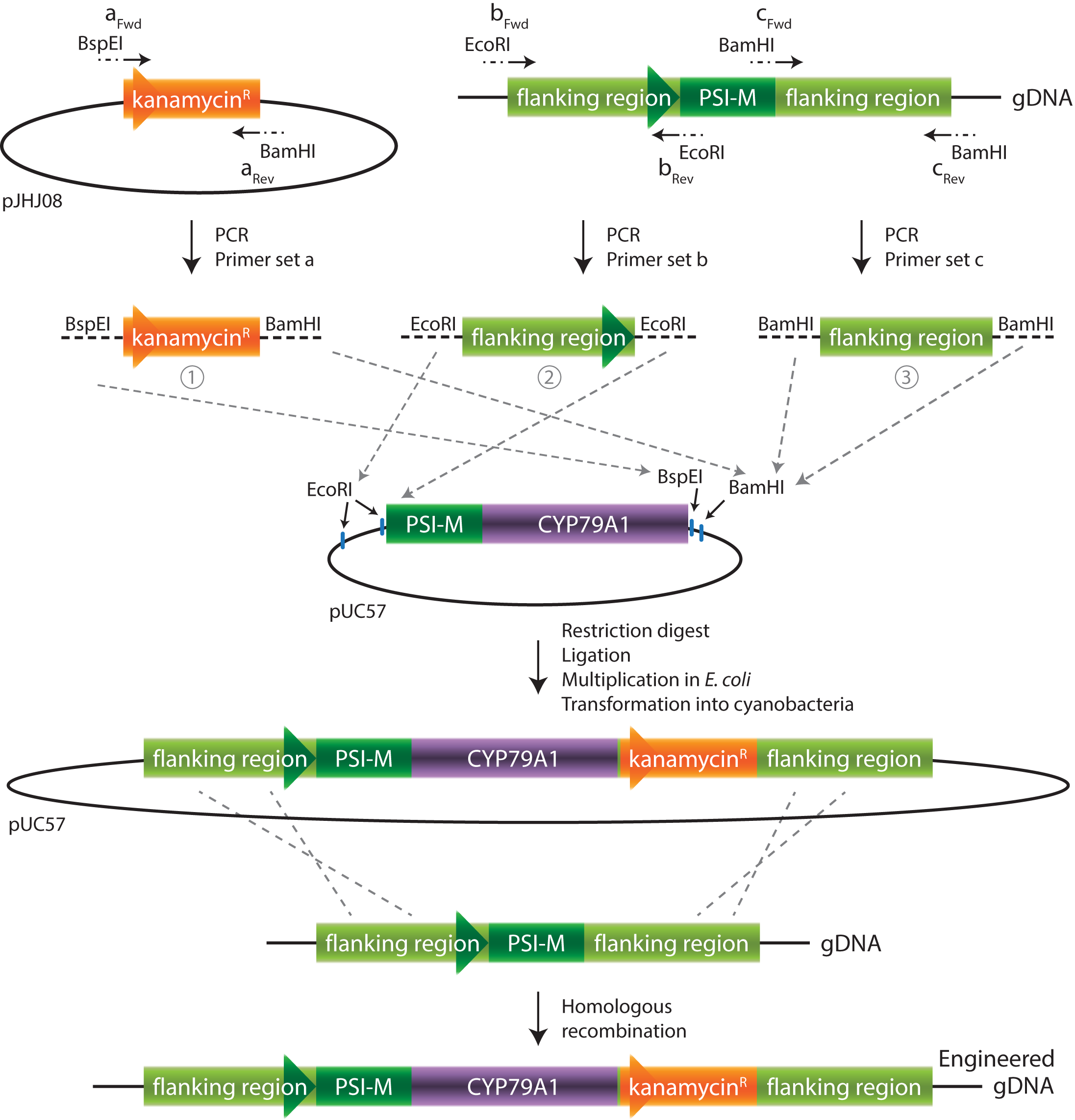

Supplement: Figure S1 — Cloning of the PsaM-CYP79A1 fusion construct. Using PCR, a kanamycin resistance cassette was amplified from a vector containing the cassette (npt cassette from pJHJ08, [25]) and flanking regions of the psaM gene were amplified from Synechococcus sp. PCC 7002 gDNA. Primer sequences are shown in Table 1. The PCR products were inserted in a commercially synthesized fusion construct of the psaM and CYP79A1 genes contained in the pUC57 cloning vector. The final construct was transformed into Synechococcus sp. PCC 7002 for replacement of the native psaM gene by homologous recombination. (TIF) [file pone.0102184.s001.tif]
